# Soil‐derived, gut‐dominant generalist bacteria shape the fitness of folivorous larvae

**DOI:** 10.1002/imt2.70169

**Published:** 2026-08-30

**Authors:** Yu‐xuan Zheng, Ying Wang, Xiao‐man Zhang, Qian Jin, Yan Zhang, Gui‐fang Wang, Yong‐qi Shao, Jing Li, Cheng Sun, Wei‐dong Kong, Cai‐qing Yang, Ai‐bing Zhang

**Affiliations:** ^1^ College of Life Sciences, Capital Normal University Beijing China; ^2^ China National Environmental Monitoring Centre Beijing China; ^3^ Research Center for Ecological Security, Ministry of Ecology and Environment Chinese Academy of Environmental Planning Beijing China; ^4^ College of Life Sciences, Hebei Normal University Hebei China; ^5^ Suqian Institute of Agricultural Sciences Jiangsu Academy of Agricultural Sciences Nanjing Jiangsu China; ^6^ Institute of Sericulture and Apiculture, College of Animal Sciences Zhejiang University Hangzhou China

**Keywords:** 16S rRNA amplicon, *Delftia* sp., Folivorous larvae, generalist bacteria, *Ralstonia insidiosa*

## Abstract

Whether Lepidoptera harbor a conserved core gut microbiome has long remained contentious. Through large‐scale microbiome profiling of folivorous larvae, their host plants, and associated soils across three climatically distinct regions of China, we identify two soil‐derived generalist bacteria, *Ralstonia insidiosa* and *Delftia* sp., that colonize 97.92% of larval species examined, attaining mean relative abundances exceeding 47%, with the soil microbial reservoir as their principal source. Strikingly, these two taxa exhibit strong mutual exclusion within the larval gut yet govern host development through diametrically opposed metabolic strategies: *R. insidiosa* promotes larval weight gain, whereas *Delftia* sp. suppresses growth. This functional bifurcation, in which two widespread generalists exert opposite phenotypic effects, represents a previously undescribed phenomenon in insect‐microbe symbiosis. Our findings provide broad evidence that soil microbial reservoirs can shape aboveground herbivore fitness via horizontally acquired bacteria, offering mechanistic insights for microbiome‐based ecological management.

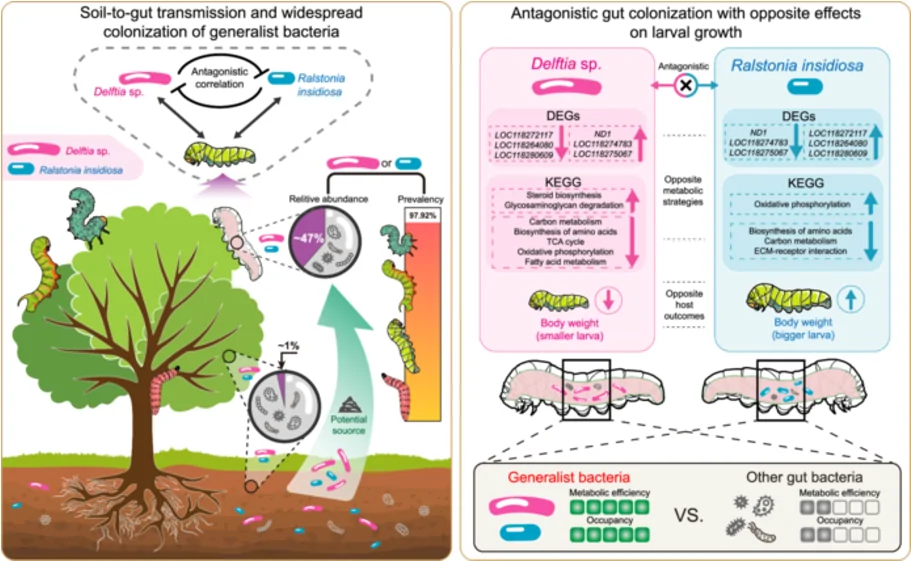

To the Editor,

Folivorous larvae, which feed primarily on leaves, play crucial roles in both natural ecosystems and human activities, yet face nutritional challenges due to leaf‐based diets that are deficient in nutrients yet rich in plant secondary metabolites (PSMs) [1, 2]. Critically, gut symbiotic bacteria are increasingly recognized as key agents facilitating PSM detoxification and nutrient acquisition [3].

Unlike social insects relying on vertically transmitted microbiomes, folivorous larvae predominantly acquire gut microbiota horizontally from environmental sources (e.g., foliage, soil) [4, 5]. This horizontal acquisition pattern has prompted suggestions that these larvae lack stable, resident microbiomes [6]. Yet this view requires re‐examination: could certain bacteria stably colonize diverse larval hosts across heterogeneous environments, conferring broad fitness advantages? Previous studies remain limited by a narrow focus on single host‐microbe interactions [7, 8], impeding the identification of environmentally sourced, cross‐host bacterial taxa of ecological significance.

To resolve this knowledge gap, we hypothesized that environmentally acquired, soil‐derived generalist bacteria exist that can: (1) stably colonize phylogenetically diverse folivorous larvae, and (2) significantly modulate host fitness. Through large‐scale field sampling across three climatic zones in China (Table S1), we profiled bacterial communities of leaf‐feeding larvae, host phyllosphere, and surrounding soils, and tracked environmental sources and gut colonization dynamics. Under natural conditions, we found, surprisingly, that diverse leaf‐feeding larvae consistently established extensive symbiotic associations with two generalist bacterial species. We further provide evidence supporting soil as a likely environmental reservoir for these two generalist bacteria, elucidated their competitive advantages within the gut trophic context, and revealed their contrasting functional roles in modulating larval body weight.

## GUT MICROBIAL DIVERSITY AND POTENTIAL SOURCES ACROSS DIVERSE FOLIVOROUS LARVAE

Leaf‐feeding larvae, host plant leaves, and topsoil were collected from Beijing (temperate continental monsoon climate), Zhejiang Province (subtropical maritime monsoon climate), and Sichuan Province (subtropical humid monsoon climate), China. Bacterial diversity in larval midguts, leaf phyllosphere, and soil samples was characterized by 16S rRNA amplicon sequencing (Figure S1A). In total, 305 leaf‐feeding larvae were collected, the majority of which were lepidopteran (*n* = 277, representing 19 families and 87 species), with the remainder comprising hymenopteran and coleopteran larvae. The dataset also included 142 host plant samples spanning 30 families and 70 species, and 106 soil samples (Table S2). Most larvae exhibited polyphagous feeding habits (Figure S1B). This broad sampling coverage enabled us to examine the general diversity patterns and potential environmental sources of larval gut microbiota under heterogeneous conditions.

The dynamics [9] and sources [10] of insect gut microbial diversity represent key dimensions for understanding insect–microbe interactions. To enable cross‐species comparisons, larval body length was normalized to relative body length (Figure S2). We observed that, with increasing relative body length, overall gut bacterial Shannon and Simpson diversity indices exhibited a gradual increasing trend (Figure S3A). Concurrently, FEAST (Fast Expectation‐Maximization for Microbial Source Tracking) ‐based exploratory source attribution suggested that the relative contribution assigned to the phyllosphere increased during larval development, whereas the relative contribution assigned to soil‐derived sources remained relatively stable (Figure S3B). As the largest microbial reservoir on Earth, soils harbor overwhelmingly higher bacterial diversity than larval guts and the phyllosphere, and this remained true even when only surface soil samples were considered (Figure S3C). Collectively, these results indicate that gut bacterial diversity and the apparent structure of environmental sources for gut bacteria of leaf‐feeding larvae change with relative body length, while the soil microbial pool may serve as a persistent and important environmental reservoir for larval gut bacteria.

### Two soil‐derived generalist bacteria, *Delftia* sp. and *ralstonia insidiosa*, extensively colonize the gut of folivorous larvae

From the collected larvae, we selected 22 species with ≥5 individuals, including 18 lepidopteran and 4 hymenopteran species. These larvae exhibited broad diets, with 17 species feeding on at least two host plant species; on average, each larval species fed on 4.18 ± 2.31 (mean ± standard deviation [SD]) plant species, spanning an average of 3.47 ± 1.90 (mean ± SD) plant genera (Figure S4A). Across these larvae, we identified 32 high‐frequency bacterial operational taxonomic units (OTUs) in the gut (with an occurrence frequency > 70% among individuals and mean relative abundance > 1%) (Figure S4B), among which OTU 4 (Burkholderiales, *Delftia* sp.) and OTU 94 (Burkholderiales, *Ralstonia insidiosa*) dominated the gut communities of most larval species. *Delftia* sp. was detected in 10 larval species, with a mean occurrence frequency of 96.30% and a mean relative abundance of 38.96%, whereas *R. insidiosa* occurred in another 10 larval species, with a mean occurrence frequency of 100% and a mean relative abundance of 34.44% (Figure S5). These two bacteria were significantly enriched in the guts of 16 larval species; notably, each larval species harbored enrichment of only one of the two taxa (Figure 1A). Together, these findings indicate that *Delftia* sp. and *R. insidiosa* can broadly adapt to gut environments shaped by different host plants and larval species, and that each larval species forms a stable and exclusive association with only one of the two bacterial taxa. At a broader scale, these two “generalist” bacteria exhibited high prevalence across the entire larval cohort (*n* = 305): 135 larvae (44.26%) harbored *Delftia* sp. at >1% relative abundance (mean = 48.02%), while 140 larvae (45.90%) harbored *R. insidiosa* at >1% relative abundance (mean = 47.73%) (Figure S6). Integrating all gut microbiome data from this study, 97.92% of larval species and 90.16% of individuals were associated with one of these two generalist bacteria, underscoring their ecological advantage and widespread symbiotic pattern in larval guts.

**FIGURE 1:**
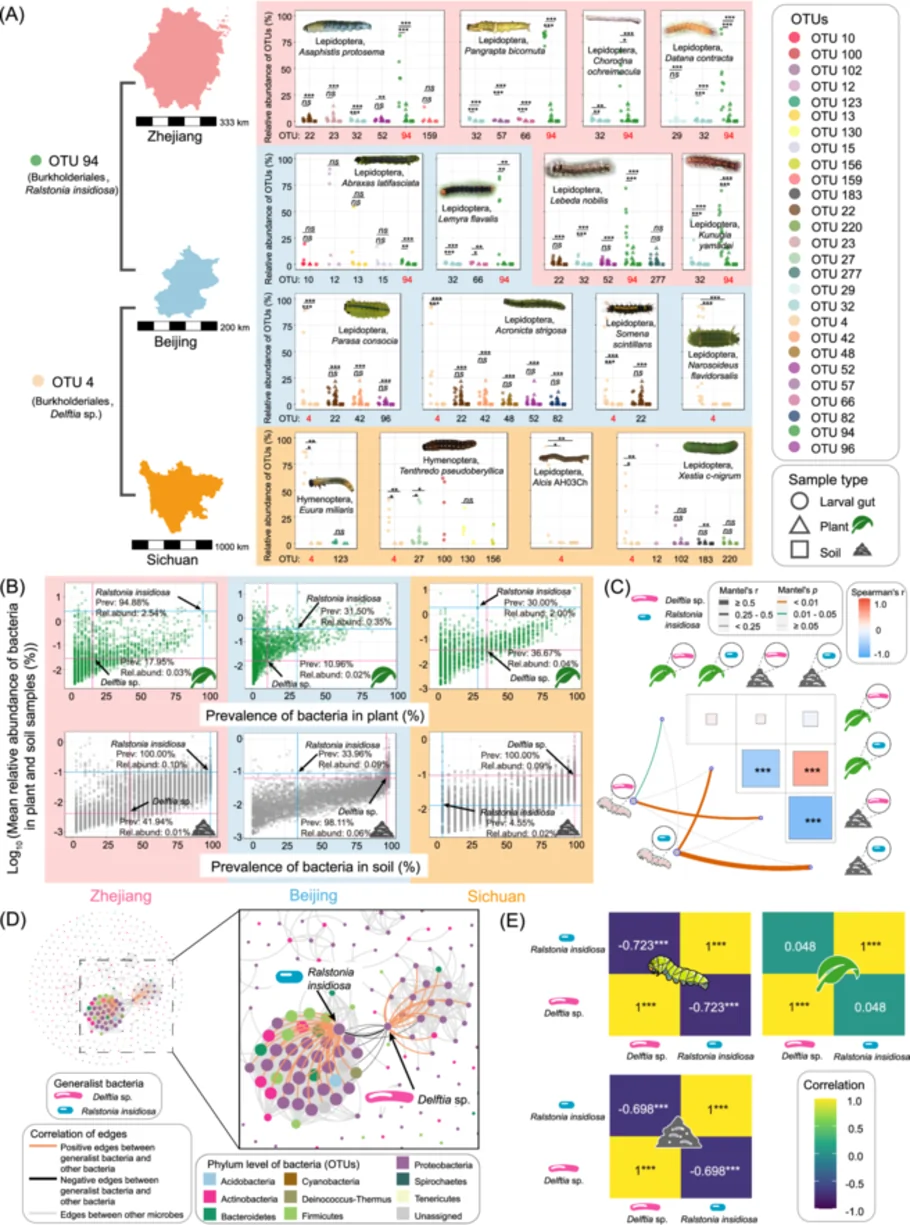
Environmental distribution patterns of the two generalist bacteria and their interactions with host larvae. (A) Comparative analysis of high‐prevalence bacterial operational taxonomic units (OTUs) between the larval gut (*n* = 143 specimens from 16 species) and environmental samples (plant phyllosphere and soil) across three provincial‐level regions in China. Symbol shape denotes microbial sample type, and color indicates bacterial OTU identity. Differences in relative abundance of the same bacterial OTU among sample types were assessed using the Wilcoxon rank‐sum test, and p values were adjusted using the Benjamini–Hochberg (BH) correction. (B) Prevalence and mean relative abundance of various bacterial OTUs in environmental samples from different provinces. In panels (A) and (B), statistical significance is indicated as follows: “ns,” not significant; “*” *p* < 0.05; “**” *p* < 0.01; “***” *p* < 0.001. (C) Pairwise comparisons of the relative abundance of the two generalist bacteria in the larval gut (*n* = 265) and environmental samples (phyllosphere, *n* = 126; soil, *n* = 106). The color gradient represents Spearman's correlation coefficient. Edge width corresponds to Mantel's r statistic for the respective distance correlations, and edge color denotes statistical significance (“***” *p* < 0.001). (D) Co‐occurrence network constructed from bacterial OTUs with a mean relative abundance >1% in the larval gut (*n* = 305), revealing a negative correlation between *Delftia* sp. and *Ralstonia insidiosa*. (E) Associations between the two generalist bacteria in larval (upper left), phyllosphere (upper right), and soil (lower left) samples. “***” *p* < 0.001.

To explore the potential environmental reservoirs of the two generalist bacteria, we analyzed OTU occurrence frequencies and mean relative abundances across all environmental samples (phyllosphere and soil) from the three regions. *Delftia* sp. was detected in 10.96%−100% of environmental samples, with a mean relative abundance of approximately 0.04%, whereas *R. insidiosa* was detected in 4.55%–100% of environmental samples, with a mean relative abundance of approximately 0.84% (Figure 1B). These results showed that at least one of the two generalist bacteria was detected in nearly all soil samples, supporting the hypothesis that larvae may acquire these bacteria from environmental reservoirs, with soils representing a likely contributor. Mantel tests further supported that variation in the two generalist bacteria in larval guts was more strongly associated with their counterparts in soil communities than with those in contemporaneously sampled phyllosphere communities (*Delftia* sp.: *r* = 0.411, *p* < 0.01; *R. insidiosa*: *r* = 0.605, *p* < 0.01) (Figure 1C). To account for the compositional nature of the 16S rRNA amplicon data, we repeated the analyses using centered log‐ratio‐transformed data. *Delftia* sp. and *R. insidiosa* remained significantly enriched in larval guts, retained a significant negative association, and showed stronger concordance with soil than with phyllosphere samples (Tables S3 and S4). A gut bacterial co‐occurrence network constructed based on all larval samples revealed a significant negative correlation between *R. insidiosa* and *Delftia* sp. (correlation coefficient = −0.723, *p* < 0.001) (Figure 1D). Notably, this negative correlation was also observed in soil microbial communities but was absent in phyllosphere communities (Figure 1E). In addition, phylogenetic signal analyses using Fritz–Purvis *D* statistic showed that the presence/absence of *Delftia* sp. (*D* = 1.325, *p* = 0.616) and *R. insidiosa* (*D* = −0.310, *p* = 0.061) were not significantly associated with host phylogeny. These results collectively suggest that the two generalist bacteria in larval guts are more closely associated with the soil microbial species pool than with the phyllosphere communities sampled at the same time, supporting soil as a likely environmental reservoir.

Regarding the potential environmental reservoirs of gut microbiota in leaf‐feeding larvae, our results are consistent with the findings of Hannula et al. [11], supporting soils as an important microbial reservoir for herbivorous insects. In addition, the dominant taxa, *Delftia* sp. and *R. insidiosa*, were more strongly associated with soil microbial communities than with the phyllosphere communities sampled at the same time. We therefore propose that these two generalist bacteria may be acquired from environmental reservoirs, particularly soil, and subsequently establish and maintain dominance within the larval gut niche.

The dominance of these two bacteria (>40% mean relative abundance in colonized larvae) across highly heterogeneous gut environments shaped by different host taxa (including Lepidoptera and Hymenoptera) and diverse plant diets highlights their exceptional ecological adaptability, consistent with their designation as true “generalists.” The strong negative association between them (*r* = −0.723) suggests intense competition or niche exclusion. Consistent with this pattern, inhibition assays using the representative *Delftia* strain, *D. tsuruhatensis* provided preliminary in vitro evidence that this strain can suppress the growth of *R. insidiosa* (Figure S7). Such competitive exclusion may arise from their overlapping nutritional requirements and metabolic niches. Notably, the tendency of hosts to be enriched with either of the two generalist bacteria exhibited no significant phylogenetic signal, further supporting the notion that this mutually exclusive pattern is more likely driven by interbacterial interactions rather than by host evolutionary history.

### Colonization of generalist bacteria in folivorous larvae and their effects on host developmental fitness

To further elucidate the potential mechanisms by which the two generalist bacteria influence host fitness in leaf‐feeding larvae, we performed metagenome functional prediction using PICRUSt2. Overall, 83.61% of larval gut microbiome samples exhibited high prediction reliability (weighted nearest‐sequenced taxon index [NSTI] < 0.06; *n* = 255), and an additional 8.85% (*n* = 27) showed moderate reliability, supporting the robustness of the functional inferences (Figure S8). Functional enrichment analyses revealed that the two generalist bacteria displayed clear metabolic advantages in larval guts, albeit along distinct specialization trajectories: 28 significantly enriched metabolic pathways were identified across eight larval species harboring *Delftia* sp., whereas 15 unique functional pathways were detected across seven larval species harboring *R. insidiosa* (all except *Lemyra flavalis* exhibited specialization) (Figure 2A). Notably, the two bacteria shared four conserved core metabolic pathways, including three closely associated with the tricarboxylic acid (TCA) cycle: 4‐hydroxyphenylacetate degradation (3‐HYDROXYPHENYLACETATE‐DEGRADATION‐PWY), nicotinamide adenine dinucleotide (NAD) biosynthesis (NADSYN‐PWY), and PWY‐5651 (Figure 2B). 4‐Hydroxyphenylacetate is a phenolic by‐product of aromatic amino acid fermentation during plant biomass degradation in animal digestive systems [12]. These pathways contribute to the generation of succinate through aromatic compound degradation, and to the production of NAD^+^ and key intermediates via tryptophan metabolism, thereby collectively sustaining efficient TCA cycle flux. Together, these results suggest that, relative to other gut bacteria, the two generalist taxa exhibit higher metabolic efficiency in the larval gut, which may enhance their ability to compete for gut niches.

**FIGURE 2:**
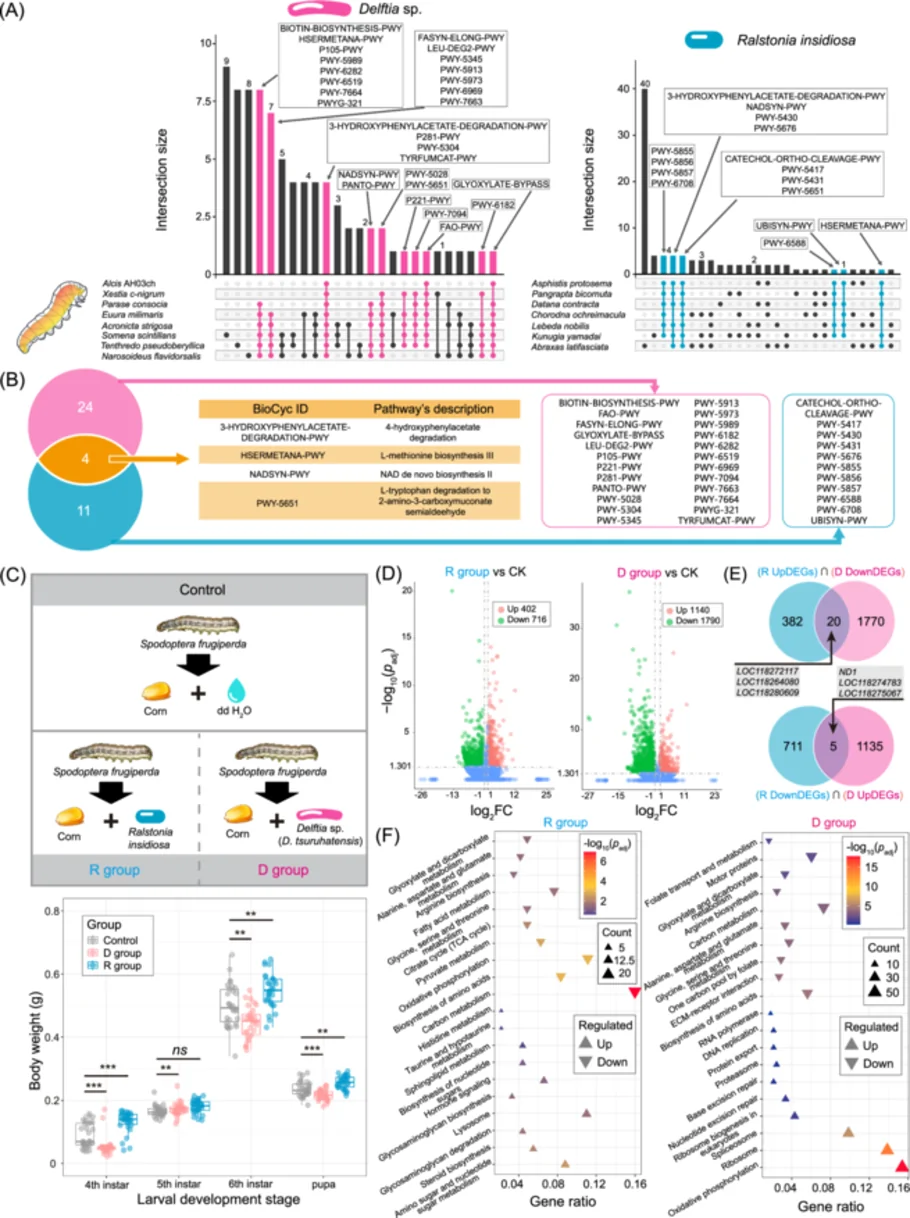
Colonization capacity of the two generalist bacteria in the gut and their effects on the performance of *Spodoptera frugiperda* larvae. (A) Functional pathways enriched by *Delftia* sp. and *Ralstonia insidiosa* relative to other gut bacteria at the MetaCyc pathway level. Pathways enriched in five or more larval species are highlighted within boxes. (B) Functional differences between pathways enriched by *Delftia* sp. (pink) and *R. insidiosa* (blue). Each pathway shown was enriched in at least five larval species. (C) Effects of *R. insidiosa* and *D. tsuruhatensis* on larval and pupal weights of *S. frugiperda*. From the fourth instar onward, larvae were continuously fed maize kernels coated with suspensions of *R. insidiosa* (R group, *n* = 30) or *D. tsuruhatensis* (D group, *n* = 30) until pupation, whereas the control group (*n* = 30) received kernels treated with ddH2O. Differences among groups were assessed using the Wilcoxon rank‐sum test, and *p* values were adjusted using the Benjamini–Hochberg (BH) correction. Statistical significance in panel (C) is indicated as follows: “*ns,*” not significant; “*” *p* < 0.05; “**” *p* < 0.01; “***” *p* < 0.001. (D) Differentially expressed genes (DEGs) identified from gut transcriptomes of larvae in the R‐ and D‐groups. (E) Genes showing opposite regulatory patterns between the R‐ and D‐groups among those identified in (D). (F) Kyoto Encyclopedia of Genes and Genomes (KEGG) enrichment analysis of larval gut transcriptomes for the R‐ and D‐groups. The top 10 significantly upregulated and top 10 downregulated KEGG pathways for each group are shown.

Bacterial transplantation assays showed that, compared with the control group, *R. insidiosa*‐colonized fall armyworm larvae (R group) exhibited significantly increased body weight at both the sixth‐instar and pupal stages. In contrast, larvae treated with the representative congeneric *Delftia* strain *D. tsuruhatensis* (D group) showed significantly reduced body weight at both stages (Figure 2C). Transcriptomic analyses further revealed host transcriptional responses associated with *R. insidiosa* and *D. tsuruhatensis* treatments. In the R group, 402 differentially expressed genes (DEGs) were upregulated and 716 were downregulated; in the D group, 1140 DEGs were upregulated and 1790 were downregulated (Figure 2D). Genes that were downregulated in the R group but upregulated in the D group included *NDI* (NADH‐ubiquinone oxidoreductase chain 1), *LOC118274783* (trypsin), and *LOC118275067* (fatty acid‐binding protein homolog 7), which are primarily involved in aerobic respiration, protein digestion, and lipid absorption and metabolism in insects. Conversely, genes that were upregulated in the R group but downregulated in the D group included *LOC118272117* (nicotinate phosphoribosyltransferase), *LOC118264080* (GPI inositol‐deacylase), and *LOC118280609* (tyrosine‐protein phosphatase 99 A), which are mainly associated with cellular energy metabolism, maintenance of cellular structural integrity, and regulation of cell proliferation (Figure 2E). Kyoto Encyclopedia of Genes and Genomes (KEGG) enrichment analyses showed that, in the R group, core biosynthetic and energy‐producing pathways—including carbon metabolism, biosynthesis of amino acids, the TCA cycle, oxidative phosphorylation, and fatty acid metabolism—were broadly downregulated, whereas steroid biosynthesis, glycosaminoglycan degradation, and amino sugar and nucleotide sugar metabolism were upregulated. In contrast, in the D group, biosynthesis of amino acids, carbon metabolism, and extracellular matrix (ECM)–receptor interaction pathways were broadly downregulated, whereas oxidative phosphorylation was upregulated (Figure 2F). Together, these results indicate that *R. insidiosa* and the representative *Delftia* strain *D. tsuruhatensis* induced contrasting body weight outcomes and host transcriptional responses in fall armyworm larvae. In this experimental context, *R. insidiosa* appears to reduce host energy expenditure, thereby reallocating conserved resources toward biomass accumulation and tissue construction. By contrast, the presence of *D. tsuruhatensis* is associated with elevated energy investment in homeostatic maintenance rather than growth; despite the upregulation of some metabolic genes, the overall metabolic state appears to be less efficient and more energetically costly, resulting in reduced body weight.

Our functional assays reveal that interbacterial competition can exert profound and opposing effects on host fitness. A possible mechanism might be that colonization by *R. insidiosa* may reduces host maintenance energy expenditure, allowing reallocating resources toward biomass accumulation and resulting in significant weight gain. In contrast, colonization by *D. tsuruhatensis* is associated with upregulation of host genes related to energy metabolism and stress responses, a state that may divert energy toward homeostatic maintenance rather than growth, ultimately resulting in weight loss. We speculate that selective enrichment of these two taxa by larvae may be context‐dependent, potentially linked to population density and resource availability. Under low‐density conditions with abundant food resources, *R. insidiosa* may facilitate enhanced nutrient accumulation and growth, whereas under high‐density conditions with limited resources, *D. tsuruhatensis* may favor reduced body mass and accelerated development toward pupation, potentially conferring resilience to food limitation and other environmental stresses. By directly linking interbacterial interactions to core host physiological phenotypes, these findings provide mechanistic insights into how gut microbiota can shape host fitness.

From a broader ecological functional perspective, members of the genera *Delftia* and *Ralstonia* have been reported to degrade a wide range of phenolic compounds and aromatic toxins [13, 14, 15, 16]. Our functional predictions are consistent with these reports and further suggest that both taxa possess efficient energy metabolic systems. Given their high abundances in larval guts, we hypothesize that, in addition to directly modulating host nutrition and metabolism, these bacteria may also indirectly enhance host tolerance to plant‐based diets by forming dense biofilm structures [17, 18], which could physically buffer gut epithelial cells from direct exposure to PSMs. This hypothesis, however, awaits direct validation by microscopic imaging and other experimental approaches.

In recent years, increasing evidence has indicated that invasive insects can rapidly adapt to novel host plants through interactions with local microbial communities [19, 20]. The two widely distributed generalist bacteria identified in this study in China—particularly the growth‐promoting *R. insidiosa*—may potentially enhance the environmental adaptability and damage potential of certain native pests or invasive insects. Accordingly, monitoring and managing key environmental microbial reservoirs may offer new avenues for developing environmentally friendly, microbiome‐informed integrated pest management strategies. Future studies should further investigate the global biogeographic distribution of these two bacteria across distinct ecoregions and employ gnotobiotic insect models to precisely dissect the molecular dialogues underlying their interactions with hosts.

In summary, through large‐scale field sampling across different climatic regions and microbiome profiling, this study systematically identified *Delftia* sp. and *Ralstonia insidiosa* as two soil‐derived, gut‐dominant generalist bacteria that are widespread in the guts of folivorous larvae. These field‐detected taxa appear to be horizontally acquired from environmental reservoirs, with soils representing a likely contributor. Functional validation further showed that *R. insidiosa* and the representative congeneric *Delftia* strain, *D. tsuruhatensis*, exerted contrasting effects on larval body weight and host metabolic pathways, suggesting that *Ralstonia*‐ and *Delftia*‐associated colonization may shape host growth and development in different directions. Together, these findings advance our understanding of the complexity of insect‐microbe interactions and highlight soil microbial reservoirs as an underappreciated but potentially pivotal factor influencing the fitness of aboveground herbivores, with implications for ecosystem management and agricultural practices.

## AUTHOR CONTRIBUTIONS

**Yu‐xuan Zheng**: Conceptualization; software; data curation; formal analysis; visualization; writing—original draft; writing—review and editing; methodology. **Ying Wang**: Conceptualization; visualization; writing—review and editing. **Xiao‐man Zhang**: Conceptualization; writing—review and editing. **Qian Jin**: Writing—review and editing. **Yan Zhang**: Methodology; visualization. **Gui‐fang Wang**: Software; formal analysis. **Yong‐qi Shao**: Writing—review and editing. **Jing Li**: Writing—review and editing. **Cheng Sun**: Writing—review and editing. **Wei‐dong Kong**: Writing—review and editing. **Cai‐qing Yang**: Conceptualization; funding acquisition; methodology; writing—review and editing. **Ai‐bing Zhang**: Conceptualization; funding acquisition; writing—review and editing.

## CONFLICT OF INTEREST STATEMENT

The authors declare no conflicts of interest.

## ETHICS STATEMENT

No vertebrate animals or human subjects were involved in this study.

## Supporting information


**Figure S1:** Sample collection and processing for bacterial 16S rRNA amplicon sequencing.
**Figure S2:** Body length standardization of lepidopteran larvae relative to the maximum body length difference between the larval and adult stages of *Spodoptera frugiperda*.
**Figure S3:** Alpha diversity and potential sources of gut bacteria in folivorous larvae.
**Figure S4:** High‐abundance bacterial OTUs in the larval gut.
**Figure S5:** Differences in the relative abundance of high‐frequency bacterial OTUs in the gut of 22 larval species.
**Figure S6:** Relative abundance distribution of *Delftia* sp. and *Ralstonia insidiosa* in the guts of larvae excluding the 16 species showing significant enrichment.
**Figure S7:** Inhibition zone assay demonstrating antagonistic interactions between *Delftia tsuruhatensis* (the representative congeneric *Delftia* strain) and *Ralstonia insidiosa*.
**Figure S8:** Accuracy evaluation of PICRUSt2 metagenome function predictions.

**Table S1:** Climate at sampling site.
**Table S2:** Sample metadata.
**Table S3:** Pairwise comparisons of the CLR‐transformed abundances of *Delftia* sp./OTU 4 and *Ralstonia insidiosa*/OTU 94 among larval gut, soil, and phyllosphere samples.
**Table S4:** Compositional‐data‐aware sensitivity analyses of the negative association and environmental concordance of *Delftia* sp. and *Ralstonia insidiosa*.
**Table S5:** Theoretical maximum larval body length.
**Table S6:** Sequence similarities among closely related species of the genera *Ralstonia* and *Delftia*, based on different 16S rRNA marker regions.

## Data Availability

The data that support the findings of this study are available in the supplementary material of this article. The 16S amplicon and RNA‐seq data can be accessed through the National Center for Biotechnology Information (NCBI) (https://www.ncbi.nlm.nih.gov/bioproject/?term=PRJNA1213266) and the Genome Sequence Archive (GSA) (https://ngdc.cncb.ac.cn/gsa/browse/CRA038465). The data and scripts used are saved in GitHub https://github.com/zhengyx1612/Zheng2026_iMeta. Supplementary materials (methods, figures, tables, graphical abstract, slides, videos, Chinese translated version and updated materials) may be found in the online DOI or iMeta Science http://www.imeta.science/.
