# Supplementary material for: Soil‐derived, gut‐dominant generalist bacteria shape the fitness of folivorous larvae
Source: Imeta. 2026 Aug 30;5(5):e70169. doi: 10.1002/imt2.70169 (PMC13527294; doi:10.1002/imt2.70169)
Supplement: Supplementary file 1 [file IMT2-5-e70169-s001.docx]

Supporting Information to

**Soil-****derived, gut-dominant generalist bacteria shape the fitness of folivorous larvae**

**Running title:** Soil-derived generalist bacteria shape larval fitness

Yu-xuan Zheng^1, 2#^, Ying Wang^1, 3#^, Xiao-man Zhang^4^, Qian Jin^5^, Yan Zhang^1^, Gui-fang Wang^1^, Yong-qi Shao^6^, Jing Li^1^, Cheng Sun^1^, Wei-dong Kong^1^, Cai-qing Yang^1*^, Ai-bing Zhang^1*^

^1^College of Life Sciences, Capital Normal University, Beijing 100048, China

^2^China National Environmental Monitoring Centre, Beijing 100012, China

^3^Research Center for Ecological Security, Ministry of Ecology and Environment, Chinese Academy of Environmental Planning, Beijing 100041, China

^4^College of Life Sciences, Hebei Normal University, Hebei 050024, China

^5^Suqian Institute of Agricultural Sciences, Jiangsu Academy of Agricultural Sciences, Jiangsu 223800, China

^6^Institute of Sericulture and Apiculture, College of Animal Sciences, Zhejiang University, Hangzhou 310058, China

^#^These authors contributed equally: Yu-xuan Zheng, Ying Wang.

*Correspondence: zhangab2008@cnu.edu.cn (Ai-bing Zhang), yangcq@cnu.edu.cn (Cai-qing Yang)

**METHODS**

**Collection of larvae, host plants, and soil samples**

In this study, folivorous larvae, host plant leaves, and soil samples were collected from Beijing (September 2019 and September 2021; 115−117°E, 39−40°N), Zhejiang (July−August 2019; 122°E, 30°N) and Sichuan (July−August 2021; 103°E, 31°N) provinces. During the day, larvae were collected from trees by the beating method [1] with supplementary sampling from shrubs and herbaceous plants conducted by visual searching. Larval specimens were stored in 2 mL, 5 mL, or 50 mL tubes filled with 100% ethanol. Simultaneously, host plant leaf samples were collected and temporarily stored in sterile plastic bags. Under aseptic conditions, leaf samples were cut into 1 cm × 1 cm pieces, placed in 50 mL sterile cryovials up to the 15 mL mark, and 20 mL of 100% ethanol was added after returning to the laboratory on the same day to preserve the samples. Soil samples were collected from the 0−10 cm topsoil layer directly beneath the plant. Three soil subsamples were randomly collected within a 3 m radius centered on the plant, thoroughly mixed to form one composite sample, and then stored in 5 mL sterile tubes. All samples (larvae, leaves, and soil) were stored at −20 ℃.

**Identification of larvae and host plant species**

All larval samples were identified by DNA barcoding, and plant samples were identified by combining DNA barcoding with morphological information. Insect genomic DNA was extracted from larval head muscle tissue using the TIANamp Genomic DNA Kit (TIANGEN). Each DNA extract was subjected to standard DNA barcoding protocols [2], and the *COI* gene [3] fragment was amplified using primer pair LCO1490 (5'-ggtcaacaaatcataaagatattgg-3') and HCO2198 (5'-taaacttcagggtgaccaaaaaatca-3'). Plant genomic DNA was extracted using the Super Plant Genomic DNA Kit (Polysaccharides & Polyphenolics-rich) (TIANGEN). Two plant barcode loci, *rbcL* [4] (F1:5'-atgtcaccacaaacagaaac-3' and 724R: 5'-tcgcatgtacctgcagtagc-3') and *trnL* [5] (A49325(c): 5'-cgaaatcggtagacgctacg-3' and B49863(d): 5'-ggggatagagggacttgaac-3'), were amplified. PCR products of insect and plant barcodes were sequenced bidirectionally on an ABI 3730XL analyzer at Beijing Zhongkexilin Biotechnology Co., Ltd.

The obtained larval barcode sequences were queried against the Barcode of Life Data Systems (BOLD) [6] database for species identification. Plant species were identified by BLAST searches of the two barcode genes against NCBI, combined with morphological comparisons to the Flora Republicae Popularis Sinicae. The larval phylogenetic tree based on the *COI* gene and the plant phylogenetic tree based on concatenated *rbcL* and *trnL* sequences were both constructed using the GTR+I+G nucleotide substitution model implemented in RAxML [7] (model parameter: RaxML -f a -x 12345 -p 12345 -# 1000 -m GTRGAMMAI -s input_aligned.fas -n nwk -k >> run.log 2>&1). The larva-host plant bipartite network was visualized using the bipartite [8] package in R.

**Normalization of Lepidopteran larval body length**

Lepidopteran larval body size varies greatly among species [9], and accurately determining the instars of field-collected larvae is extremely difficult. This variability complicates direct comparisons of gut microbial diversity across different lepidopteran taxa. To facilitate such comparisons, we standardized larval body length using a straightforward approach, allowing approximate assessment of how gut microbial community composition varies with development across different lepidopteran larvae. We measured the maximum body length of laboratory-reared *Spodoptera frugiperda* (Lepidoptera: Noctuidae) (*SF*) larvae (*n* = 37) and the body length of newly emerged adults (*n* = 40), and calculated the ratio of maximum larval body length to adult body length, *i.e*:

$\text{Ratio}_{\frac{\text{Larva}}{\text{Adult}}}\text{=}\frac{{\text{Larval}\text{\_}\text{maximum}\text{\_}\text{body}\text{\_}\text{lengt}\text{h}}_{\text{SF}}}{{\text{Adult}\text{\_}\text{body}\text{\_}\text{length}}_{\text{SF}}}\text{=1.92}$.

Subsequently, for each species, the theoretical maximum larval body length was estimated as:

$\text{Larval}\text{\_}\text{t}\text{h}\text{eoretical}\text{\_}\text{maximum}\text{\_}\text{body}\text{\_}\text{lengt}\text{h}\text{=}\text{Ratio}_{\frac{\text{Larva}}{\text{Adult}}}\text{×}\text{Adult}\text{\_}\text{body}\text{\_}\text{length}$,

Adult body length data were obtained from scaled photographs in the BOLD database. Subsequently, the body length of each larva was standardized as:

$\text{Normalized}\text{\_}\text{larval}\text{\_}\text{length}\text{=}$ $\frac{\text{Actual}\text{\_}\text{larval}\text{\_}\text{body}\text{\_}\text{lengt}\text{h}}{\text{Larval}\text{\_}\text{t}\text{h}\text{eoretical}\text{\_}\text{maximum}\text{\_}\text{body}\text{\_}\text{lengt}\text{h}}\text{×100\%}$,

If the calculated value exceeded 100%, it was capped at 100%. For lepidopteran species lacking scaled adult photographs in BOLD Systems, photographs of closely related species in the same genus or family were used as substitutes, and the adult body length was replaced by the mean body length of all available related species. The theoretical maximum larval body lengths and the data for related species used in this study are provided in Table S5.

**16S rRNA amplicon sequencing**

Larval midguts were dissected on a clean bench. Each larva was washed in sterile water for 1 min before dissection to remove surface microorganisms, and the sterile water was replaced after each wash. Approximately 200 mg of midgut tissue was excised, placed directly into a 1.5 mL sterile centrifuge tube, and flash-frozen in liquid nitrogen for subsequent microbial DNA extraction. For larvae with a body length < 1 cm from which the midgut could not be completely separated, the whole larva was washed twice in sterile water (30 s each time, with water replaced between washes). After washing, the midsection of the larva was dissected, transferred to a 1.5 mL sterile centrifuge tube, and flash-frozen in liquid nitrogen prior to gut microbial DNA extraction.

The 50 mL cryovial containing leaf fragments and 20 mL of 100% ethanol was shaken at 200 rpm for 30 min. The 15 mL of ethanol was then immediately transferred to a 15 mL sterile centrifuge tube and centrifuged at 10,000 rpm for 15 min. The ethanol was carefully decanted, and the pellet was retained for phyllosphere microbial DNA extraction. Approximately 200 mg of soil was used for soil microbial DNA extraction. Genomic DNA from larval gut, phyllosphere, and soil microbiota was extracted using the MolPure Soil DNA Kit (YEASEN Biotech Co., Ltd., Shanghai, China).

The V3−V4 region of 16S rRNA gene was amplified by PCR using primers 338F [10] (5'-actcctacgggaggcagcag-3') and 806R (5'-ggactachvgggtwtctaat-3'). A negative control was included in each PCR batch. Amplicons were sequenced on the Illumina MiSeq platform using the paired-end 300-bp (PE300) method (Majorbio, Shanghai, China).

Raw sequencing data were processed using the EasyAmplicon pipeline [11] for bacterial species annotation. Key parameters were as follows: after primer removal, raw reads were quality-filtered using VSEARCH with the option --fastq_maxee_rate 0.01, limiting the maximum expected error rate to 1% per base. Identical sequences were collapsed with --derep_fulllength, and low-abundance sequences were removed by setting --minuniquesize 100, retaining only sequences represented by at least 100 reads to minimize potential sequencing artefacts. OTUs were clustered at 97% similarity. Taxonomic annotation was performed using the RDP database [12]. After removal of chloroplast-derived sequences, three alpha diversity indices (richness, Simpson, and Shannon) were calculated using USEARCH and VSEARCH. The Simpson index was expressed as 1-D.

**Microbiota source tracking analysis**

Microbial source tracking was performed using the FEAST [13] package in R. For each larva, the gut microbiota was designated as the sink, and the microbiota of the host plant phyllosphere and soil were designated as sources. Source tracking for each gut microbial sample was independently repeated 100 times, and the mean contribution proportions were calculated. This yielded three source proportion estimates per larva—plant-derived, soil-derived, and unknown—which summed to 1. Because only the phyllosphere and soil samples collected at the time of larval sampling were included as source communities, FEAST results were interpreted as exploratory source attribution among the sampled source categories rather than exhaustive reconstruction of all microbial sources encountered by larvae throughout development.

**Analysis of correlations of larval gut bacterial diversity and source proportions with larval body length**

All statistical analyses were performed in R using the glmmTMB package [14]. These analyses included 239 larval samples selected from the full larval dataset for which both relative body length and source attribution data were available. Generalized linear mixed models (GLMMs) were constructed to examine relationships between larval body length and six gut microbial response variables: OTU richness, Shannon diversity, Simpson diversity, and proportions of soil-derived, phyllosphere-derived, and unknown source taxa. Based on distributional assessment, OTU richness was modeled with a negative binomial distribution (log link), Shannon diversity with a Gaussian distribution, and Simpson index and source proportions with a beta distribution (logit link); boundary values (0 or 1) were adjusted by ± 0.001.

To account for the hierarchical data structure, we compared four random effect structures using Akaike information criterion (AIC): no random effects, insect family intercept, species nested within family, and species nested within family plus plant family intercept; the structure with the lowest AIC was selected. For each initial model, a quadratic term for body length was tested via likelihood ratio tests. As all were non-significant (*p* > 0.05), models were simplified to include only the linear body length term with the chosen random structure.

Model validation was conducted using the DHARMa [15] package: residuals were simulated and tested for overdispersion and zero-inflation. Marginal *R*^2^ (fixed effects only) was computed with the performance package. Predictions were generated for fixed effects only, with 95% confidence intervals on the response scale (back-transformed via inverse logit for beta models).

**Microbial enrichment analysis and correlation analysis of generalist bacteria**

The Wilcoxon rank-sum test was used to compare the relative abundances of microbial components between larval gut and environmental samples (phyllosphere and soil). The Mantel test was employed to examine the correlation between the compositional dissimilarities of *Ralstonia insidiosa* and *Delftia* sp. in the larval gut and those in the paired environmental samples (leaf surfaces and soil). This Mantel test was based on Spearman’s rank correlation and included only paired environmental samples. These analyses were performed using the linkET [16] and dplyr [17] packages in R. To account for the compositional nature of 16S rRNA amplicon data, zero replacement and centered log-ratio (CLR) transformation were applied using the zCompositions [18] and compositions [19] packages in R, respectively. The CLR-transformed abundances of *Delftia* sp. and *R. insidiosa* were subsequently extracted. Their enrichment in larval gut, soil, and phyllosphere samples was re-evaluated by pairwise Wilcoxon rank-sum tests with Benjamini-Hochberg correction. The association between the two bacteria in larval gut samples was further evaluated using Spearman’s rank correlation of their CLR-transformed abundances and Fisher’s exact tests on presence/absence and dominance status (derived from raw counts). Euclidean distance matrices were calculated from the CLR-transformed abundances. The distance matrix for larval gut samples was compared with those of paired soil and paired phyllosphere samples using Spearman’s Mantel tests with 9999 permutations, as implemented in the vegan [20] package. To explore whether larval phylogeny influences the enrichment of specific bacteria, we used the Fritz-Purvis *D* statistic [21] to test for phylogenetic signal in the presence/absence of two bacterial taxa (*Delftia* sp. and *R. insidiosa*). The *D* statistic compares the observed distribution of binary traits to random (*D* = 1) and Brownian threshold model (*D* = 0) expectations, with significance assessed via 1000 permutations.

**Microbial co-occurrence network**

The Psych [22] package in R was used to calculate Spearman correlations between bacterial OTUs (average relative abundance ≥ 1% in each sample). The significantly related bacterial OTUs (the correlation between each bacterial OTU was significant than 0.6, and the *p*-value was less than 0.05) were visualized by Gephi 0.10.1 (https://gephi.org/).

***In vitro* antagonism assay between the representative *Delftia* candidate strain *D. tsuruhatensis* and *Ralstonia insidiosa***

The resolution of 16S rRNA amplicon sequencing is limited for species-level identification within some bacterial genera, including *Delftia*. Therefore, the field-detected OTU 4 was conservatively annotated as *Delftia* sp. rather than assigned to a single species. To select candidate strains for functional validation, we compared the OTU 4 sequence with publicly available sequences of described *Delftia* species. Within the V3−V4 region amplified by the 338F/806R primer pair used in this study, *D. lacustris* and *D. tsuruhatensis* both showed 100% sequence similarity to OTU 4 and were therefore selected as candidate taxa for preliminary assays (Table S6). Because our aim was to test whether an OTU 4-related *Delftia* candidate could colonize the larval gut and influence host development without causing direct mortality, we first performed preliminary feeding assays. Under our experimental conditions, *D. lacustris* caused larval mortality, whereas *D. tsuruhatensis* colonized the larval gut without lethal effects. We therefore used *D. tsuruhatensis* CGMCC 1.8821 as a representative congeneric candidate strain in subsequent larval body-weight and transcriptomic experiments.

*D*. *tsuruhatensis* and *R. insidiosa* were cultured separately in LB broth at a constant temperature to a final density of 10^8^ CFU/mL. Each culture was divided into two aliquots: one aliquot was centrifuged at 12,000 rpm, and the supernatant was collected and sterilized. The resulting *D. tsuruhatensis* supernatant, *D. tsuruhatensis* cell suspension, *R. insidiosa* supernatant, and *R. insidiosa* cell suspension were each mixed with LB agar cooled to ~50 ℃ to prepare mixed agar plates. LB agar plates without added bacterial cells or supernatants served as controls. On each mixed plate, two sterile filter paper discs were placed, and 40 μL of the heterologous bacterial suspension (the species not incorporated into the agar) and ddH_2_O were added to the two discs, respectively. For control plates, three sterile discs were placed and loaded with each bacterial suspension and ddH_2_O separately. All treatments were performed in triplicate, and inhibition zones were recorded at 0, 24, 48, 72, and 96 h.

**Prediction of metagenome functions**

PICRUSt2 [23] was used to infer metagenomic functional profiles from the 16S rRNA amplicon data at the MetaCyc pathway level. The pathway contributions of the two focal generalist bacteria, *Delftia* sp. and *Ralstonia insidiosa*, were evaluated separately for each larval species using edgeR [24] package in R. For each analysis, a MetaCyc pathway contribution count matrix was constructed, in which each row represented a MetaCyc pathway and each column represented an OTU-level pathway contribution profile. The design matrix included bacterial group as the sole fixed effect, comparing the focal generalist bacterium (*Delftia* sp. or *R. insidiosa*) with the other bacterial OTUs detected in the same larval species. Differential pathway contributions were assessed using the negative-binomial generalized linear model framework implemented in edgeR. Count matrices, including zero counts, were analyzed directly without zero replacement or pseudocount addition. *p*-values were adjusted across all MetaCyc pathways tested within each comparison using the Benjamini-Hochberg procedure to control the false discovery rate (FDR). Pathways with a positive log_2_ fold change, nominal *p* < 0.05, and FDR < 0.20 were considered significantly enriched in the focal generalist bacterium, whereas pathways with a negative log_2_ fold change meeting the same significance criteria were considered depleted.

**Effects of dietary supplementation with *Ralstonia insidiosa* or *Delftia sp.* (represented by the congeneric strain *D. tsuruhatensis*) on larval development**

To investigate whether the gut generalist bacteria influence host growth and development, *S. frugiperda* larvae were fed corn kernels coated with *R. insidiosa* CGMCC 1.3145 (R group) or *D. tsuruhatensis* CGMCC 1.8821 (D group). *S. frugiperda* larvae were reared on corn kernels until the third instar. At the onset of the fourth instar, larvae were randomly assigned to three groups: R group (inoculated with *R. insidiosa*, *n* = 30), D group (inoculated with *D. tsuruhatensis*, *n* = 30), and control group (CK, inoculated with sterile water, *n* = 30). Bacterial strains were cultured in LB broth at a constant temperature to an OD_600_ of 1.0, centrifuged at 6000 rpm, and resuspended in an equal volume of sterile water. The resulting suspensions were used to inoculate corn kernels for larval feeding; the control group received sterile water only. Corn kernels were replaced daily, and larval body weight was recorded daily.

To evaluate treatment effects on larval growth, differences in body weight among groups were compared using the Wilcoxon rank-sum test, as the data deviated from normality. Subsequently, from each treatment group, three sixth-instar larvae were randomly selected, and their midguts were pooled as one composite sample. Four such composite samples (*n* = 4) per group were prepared for transcriptomic analysis.

**Total RNA extraction and transcriptome sequencing**

First, place the larval midgut tissues directly into a mortar, add a small amount of liquid nitrogen, and grind rapidly. Then, transfer the ground tissue sample into a centrifuge tube at a ratio of 1 mL of Trizol reagent per 50−100 mg of tissue. Add chloroform at a volume equivalent to 1/5 of the total mixture, shake vigorously for 15 s, and incubate at room temperature for 2−3 min.Centrifuge the mixture at 12,000 rpm and 4 ℃ for 10 min. Carefully aspirate the upper aqueous phase into a new centrifuge tube, and add an equal volume of isopropanol. Invert the tube gently to mix thoroughly, then incubate at room temperature for 10 min. Centrifuge again at 12,000 rpm and 4 ℃ for 10 min. Discard the supernatant, and wash the precipitate thoroughly with 1 mL of 75% ethanol prepared with DEPC-treated water. Centrifuge at 12,000 rpm and 4 ℃ for 3 min, discard the supernatant, and air-dry the precipitate at room temperature for 2−3 min. Add an appropriate volume of RNase-free water (DEPC-treated water) to dissolve the precipitate completely, then conduct RNA quality assessment. Qualified RNA samples were used for cDNA library construction, with subsequent transcriptome sequencing performed by Novogene Co., Ltd. (Beijing, China).

The raw sequencing data were quality-controlled using fastp to obtain clean reads. These clean reads were then aligned to the reference genome using HISAT2 [25]. Gene expression levels were quantified using feature Counts. Differentially expressed genes (DEGs) were identified with the thresholds of an adjusted *p*-value (*p*_adj_) ≤ 0.05 and an absolute log_2_ fold change ≥ 1. Functional enrichment analysis of the DEGs was performed based on the KEGG database. A KEGG pathway was considered significantly enriched if its corrected *p*-value was less than 0.05.

**REFERENCES**

1.	Wang, Ming-Qiang, Yi Li, Douglas Chesters, Helge Bruelheide, Keping Ma, Peng-Fei Guo, Qing-Song Zhou, et al. 2020. “Host functional and phylogenetic composition rather than host diversity structure plant-herbivore networks.” *Molecular Ecology* 29: 2747–62. https://doi.org/10.1111/mec.15518

2.	Shi, Zhi-yong, Cai-qing Yang, Meng-di Hao, Xiao-yang Wang, Robert D. Ward and Ai-bing Zhang. 2018. “FuzzyID2: A software package for large data set species identification via barcoding and metabarcoding using hidden Markov models and fuzzy set methods.” *Molecular Ecology Resources* 18: 666–75. https://doi.org/10.1111/1755-0998.12738

3.	Folmer, Ole, Mb Black, Hoeh Wr, R. Lutz and Robert C Vrijenhoek. 1994. “DNA primers for amplification of mitochondrial cytochrome c oxidase subunit I from diverse metazoan invertebrates.” *Molecular Marine Biology and Biotechnology* 3: 294–9.

4.	Lledό, M. Dolores, Manuel B. Crespo, Kenneth M. Cameron, Michael F. Fay and Mark W. Chase. 1998. “Systematics of Plumbaginaceae based upon cladistic analysis of rbcl sequence data.” *Systematic Botany* 23: 21–9. https://doi.org/10.2307/2419571

5.	Taberlet, Pierre, Eric Coissac, Francois Pompanon, Ludovic Gielly, Christian Miquel, Alice Valentini, Thierry Vermat, et al. 2007. “Power and limitations of the chloroplast trnl (UAA) intron for plant DNA barcoding.” *Nucleic Acids Research* 35: e14. https://doi.org/10.1093/nar/gkl938

6.	Ratnasingham, Sujeevan and Paul D. N. Hebert. 2007. “BOLD: The Barcode of Life Data System (www.barcodinglife.org).” *Molecular Ecology Notes* 7: 355–64. https://doi.org/10.1111/j.1471-8286.2007.01678.x

7.	Stamatakis, Alexandros. 2006. “RAxML-VI-HPC: maximum likelihood-based phylogenetic analyses with thousands of taxa and mixed models.” *Bioinformatics* 22: 2688–90. https://doi.org/10.1093/bioinformatics/btl446

8.	Dormann Carsten F, Bernd Gruber, and Jochen Fründ. 2008. “Introducing the bipartite package: Analysing ecological networks.” *R News* 8(2): 8−11.

9.	Albaugh, David, Bob Cammarata, Ros Field, Harold Greeney and Kirby Wolfe. 2017. “*The Book of Caterpillars*.”

10.	Xu, Nan, Guangcai Tan, Hongyuan Wang and Xiapu Gai. 2016. “Effect of biochar additions to soil on nitrogen leaching, microbial biomass and bacterial community structure.” *European Journal of Soil Biology* 74: 1–8. https://doi.org/10.1016/j.ejsobi.2016.02.004

11.	Liu, Yong-Xin, Lei Chen, Tengfei Ma, Xiaofang Li, Maosheng Zheng, Xin Zhou, Liang Chen, et al. 2023. “EasyAmplicon: An easy-to-use, open-source, reproducible, and community-based pipeline for amplicon data analysis in microbiome research.” *iMeta* 2: e83. https://doi.org/10.1002/imt2.83

12.	Maidak, Bonnie L., Niels Larsen, Michael J. Mccaughey, Ross Overbeek, Gary J. Olsen, Karl Fogel, James Blandy, et al. 1994. “The ribosomal database project.” *Nucleic Acids Research* 22: 3485–7. https://doi.org/10.1093/nar/22.17.3485

13.	Shenhav, Liat, Mike Thompson, Tyler A. Joseph, Leah Briscoe, Ori Furman, David Bogumil, Itzhak Mizrahi, et al. 2019. “FEAST: fast expectation-maximization for microbial source tracking.” *Nature Methods* 16: 627–32. https://doi.org/10.1038/s41592-019-0431-x

14.	McGillycuddy, Maeve, Benjamin M. Bolker, Gordana Popovic and David I. Warton. 2025. “Parsimoniously fitting large multivariate random effects in glmmTMB.” *Journal of Statistical Software* 112: 1–19. https://doi.org/10.18637/jss.v112.i01

15.	Hartig, Florian, Lukas Lohse, Melina de Souza Leite and Cosmina Werneke. 2024. “DHARMa: Residual diagnostics for hierarchical (Multi-Level / Mixed) regression models.”

16.	Huang, Houyun. 2021. “linkET: Everything is Linkable.” R package version 0.0.3. https://github.com/Hy4m/linkET

17.	Wickham, Hadley and Romain François. 2014. “dplyr: A grammar of data manipulation.” R package version 0.3.

18.	Palarea-Albaladejo, Javier and J. Antoni Martín-Fernández. 2015. “zCompositions - R Package for multivariate imputation of left-censored data under a compositional approach.” *Chemometrics and Intelligent Laboratory Systems* 143: 85–96. https://doi.org/10.1016/j.chemolab.2015.02.019

19.	Boogaart, K. Gerald van den, Raimon Tolosana-Delgado and Matevz Bren. 2025. “compositions: Compositional data analysis.” R package version 2.0-9. https://CRAN.R-project.org/package=compositions

20.	Dixon, Philip. 2003. “VEGAN, a package of R functions for community ecology.” *Journal of Vegetation Science* 14: 927–30. https://doi.org/10.1111/j.1654-1103.2003.tb02228.x

21.	Susanne A. Fritz and Andy Purvis. 2010. “Selectivity in mammalian extinction risk and threat types: a new measure of phylogenetic signal strength in binary traits.” *Conservation Biology* 24: 1042-51. https://doi.org/10.1111/j.1523-1739.2010.01455.x

22.	Revelle, William. 2011. “psych: Procedures for psychological, psychometric, and personality research.” R Package Version 1.0–95.

23.	Douglas, Gavin M., Vincent J. Maffei, Jesse R. Zaneveld, Svetlana N. Yurgel, James R. Brown, Christopher M. Taylor, Curtis Huttenhower, et al. 2020. “PICRUSt2 for prediction of metagenome functions.” *Nature Biotechnology* 38: 685–8. https://doi.org/10.1038/s41587-020-0548-6

24.	Robinson, Mark D., Davis J. McCarthy and Gordon K. Smyth. 2010. “edgeR: A bioconductor package for differential expression analysis of digital gene expression data.” *Bioinformatics* 26: 139–40. https://doi.org/10.1093/bioinformatics/btp616

25.	Kim, Daehwan, Ben Langmead and Steven L. Salzberg. 2015. “HISAT: a fast spliced aligner with low memory requirements.” *Nature Methods* 12: 357–60. https://doi.org/10.1038/nmeth.3317

**Figure S1 Sample collection and processing for bacterial 16S rRNA amplicon sequencing.** (A) Study sites and sampling distribution. Field collections were conducted across three ecologically distinct provincial-level regions in China: Beijing, Zhejiang, and Sichuan. The sampling strategy encompassed three key components: folivorous larvae, host plant leaves, and soil. The proportional distribution of collected samples across provinces is quantitatively represented in the pie chart. Larvae were collected from plants by the beating method, and leaves of host plants and shallow soil under the vegetation were collected simultaneously. Each larva was stored separately; five leaves were randomly collected per plant, and soil samples were obtained by randomly sampling three locations within a 3-meter radius of the plant and pooling them into one composite sample. All samples were transported to the laboratory for bacterial 16S rRNA amplicon sequencing. The base map of China was obtained from the National Platform for Common Geospatial Information Services (Tianditu, http://www.tianditu.gov.cn), approval number: GS(2019)6379. (B) Larval feeding network. The larval phylogenetic tree (bottom half) is based on *COI* sequences, and the host plant phylogenetic tree (left half) is based on *rbcL* and *trnL* sequences. The sample size of each larval species (displayed only for species with ≥ 3 individuals) and the species identification similarity based on BOLDSYSTEMS (displayed only for BOLD Similarity ≥ 98%) are labeled at the branch tips; the sample size of plants (displayed only for species with ≥ 3 individuals) is also indicated.

**Figure S2 Body length standardization of lepidopteran larvae relative to the maximum body length difference between the larval and adult stages of *Spodoptera frugiperda*.**

**Figure S3 Alpha diversity and potential sources of gut bacteria in folivorous larvae.** (A) Relationships between gut bacterial alpha diversity (*n* = 239) and the relative body length of leaf-feeding lepidopteran larvae. (B) Relationships between the inferred source proportions of gut bacteria (*n* = 239) and the relative body length of leaf-feeding lepidopteran larvae. Significance was evaluated at α = 0.05 (two-tailed). (C) 16S rRNA amplicon sequencing results and bacterial alpha diversity in larval gut samples (*n* = 305), plant phyllosphere samples (*n* = 142), and soil samples (*n* = 106). In panels (A) and (B), statistical significance is indicated as follows: “*ns*”, not significant; “*” *p* < 0.05; “**” *p* < 0.01; “***” *p* < 0.001.

**Figure S4 High-abundance bacterial OTUs in the larval gut.** (A) Body length distribution and host plant composition of larvae (22 larval species with ≥ 5 individual replicates, *n* = 179). Node color indicates the host plant of different larval individuals, and the black bar represents the theoretical maximum body length of the given larval species. (B) Occurrence frequency (> 70%) and mean relative abundance (> 1%) of high-frequency OTUs in the gut of different larval species.

**Figure S5 Differences in the relative abundance of high-frequency bacterial OTUs in the gut of 22 larval species.** Relative abundance of each high-frequency bacterial OTU was compared between gut samples from 22 larval species (each represented by at least five individuals, *n* = 179) and the corresponding environmental samples (host plants and soil). Point shape indicates the source of microbial samples, and color represents distinct bacterial OTUs. Plant and soil samples were selected from the provinces and cities where larvae were collected and include all samples containing the corresponding OTUs. Differences in relative abundance of the same bacterial OTU among samples were assessed using the Wilcoxon rank-sum test, and *p*-values were adjusted using the Benjamini-Hochberg (BH) correction. Statistical significance is indicated as follows: “*ns*”, not significant; “*” *p* < 0.05; “**” *p* < 0.01; “***” *p* < 0.001.

**Figure S6 Relative abundance distribution of *Delftia* sp. and *Ralstonia insidiosa* in the guts of larvae excluding the 16 species showing significant enrichment.** After excluding 16 larval species that showed significant enrichment, the analysis included the remaining individuals (*n* = 162), which comprised six larval species without statistical significance in Figure S5 and those represented by fewer than five replicates. The Sankey diagram categorizes larvae based on a 1% relative abundance threshold for the generalist bacteria. Boxplots show the relative abundance distribution within larvae that harbor each bacterium above this threshold.

**Figure S7 Inhibition zone assay demonstrating antagonistic interactions between *Delftia tsuruhatensis* (the representative congeneric *Delftia* strain) and *Ralstonia insidiosa*.** Five experimental groups were established. Group 01: Filter paper discs loaded with *R. insidiosa* suspension or ddH_2_O (CK) were placed on LB agar plates pre-mixed with *D. tsuruhatensis* culture supernatant. Group 02: Filter paper discs loaded with *D. tsuruhatensis* suspension or ddH_2_O (CK) were placed on LB agar plates pre-mixed with *R. insidiosa* culture supernatant. Group 03: Filter paper discs loaded with *R. insidiosa* suspension or ddH_2_O (CK) were placed on LB agar plates pre-mixed with *D. tsuruhatensis* bacterial suspension. Group 04: Filter paper discs loaded with *D. tsuruhatensis* suspension or ddH_2_O (CK) were placed on LB agar plates pre-mixed with *R. insidiosa* bacterial suspension. Group 05: Filter paper discs loaded with *R. insidiosa* suspension, *D. tsuruhatensis* suspension, or ddH_2_O (CK) were placed on LB agar plates without any pre-mixed bacteria.

**Figure S8 Accuracy evaluation of PICRUSt2 metagenome function predictions.** Numbers in the bar chart indicate the sample size of each component.
